# Furin Overexpression Suppresses Tumor Growth and Predicts a Better Postoperative Disease-Free Survival in Hepatocellular Carcinoma

**DOI:** 10.1371/journal.pone.0040738

**Published:** 2012-07-10

**Authors:** Ya-Hui Huang, Kwang-Huei Lin, Chen-Hsin Liao, Ming-Wei Lai, Yi-Hsin Tseng, Chau-Ting Yeh

**Affiliations:** 1 Department of Hepato-Gastroenterology, Liver Research Center, Chang Gung Memorial Hospital, Taoyuan, Taiwan; 2 Department of Biochemistry, School of Medicine, Chang Gung University, Taoyuan, Taiwan; 3 Department of Pediatric Gastroenterology, Chang Gung Children Hospital, Taoyuan, Taiwan; 4 Molecular Medicine Research Center, Chang Gung University, Taoyuan, Taiwan; Broad Institute of Massachusetts Institute of Technology and Harvard University, United States of America

## Abstract

Furin is a member of the pro-protein convertase family. It processes several growth regulatory proteins into their active forms, which are critical to tumor progression, metastasis, and angiogenesis. Furin over-expression could occur in liver cancer and a previous study showed that over-expression of furin promoted HepG2 cell invasion in tail vein xenograft models. However, the clinical relevance of furin expression in hepatocellular carcinoma (HCC) remained unknown. Surprisingly, in a postoperative survival analysis for HCC patients, it was found that the tumor/non-tumor (T/N) ratio of furin expression ≥ 3.5 in HCC tissues predicted a better postoperative disease-free survival (DFS) (*P* = 0.010; log-rank test). Furthermore, subcutaneous xenograft experiments demonstrated a significant suppression effect of tumor growth in the furin-overexpressed xenografts (Huh7-Furin) compared to the mock control. Administration of a synthetic furin inhibitor for inhibition of the pro-protein convertase activity, decanoyl-Arg-Val-Lys-Arg-chloromethylketone (decRVKR-CMK), to the Huh7-Furin xenograft bearing mice restored the repression effect of tumor growth. In contrast, administration of decRVKR-CMK to the mock Huh7 xenograft bearing mice showed no change in growth rate. In conclusion, furin overexpression inhibited HCC tumor growth in a subcutaneous xenograft model and predicted a better postoperative DFS in clinical analysis.

## Introduction

Furin, a member of the pro-protein convertase (PC) family, activates precursor proteins by cleavage of the specific recognition sequence RXK/PR during the transport through the Golgi/*trans*-Golgi secretory pathway [1]–[3]. Furin and other PC family members process several latent precursor proteins into mature active products, including growth factors, hormones, receptors, plasma proteins, and matrix metalloproteases (MMPs). These proteins are critical to the proper physiological function in cells [1], [2], [4]. Most of the functions of furin substrates are related to cancer cell growth, such as tumor progression, metastasis, and angiogenesis [5]–[7].

Numerous studies have reported that furin overexpression occurred in cancers of various organs including ovary [8], breast [9], head and neck [10], lung [11], [12], and brain [13]. Several lines of evidence suggest that strategies of furin inhibition can be implemented to anti-cancer therapy [7], [13]–[16]. Both decanoyl-Arg-Val-Lys-Arg-chloromethylketone (decRVKR-CMK) and α1-antitrypsin Portland (α1-PDX) have been used as a furin inhibitor. Treatment of head and neck squamous cell carcinoma (HNSCC) cells with decRVKR-CMK decreases substrate activation and thus cell proliferation rate and invasiveness [17]. Administration of the α1-PDX suppressed cell growth and invasiveness in glioma tumor or HNSCC cell lines [13], [14].

Previously, it was reported that overexpression of furin occurred in hepatocellular carcinoma (HCC) and furin-overexpressed HepG2 cells promoted their invasion ability in an animal model [18]. In view of the similar effect of furin in hepatoma cells compared to other types of cancers, furin might serve as a candidate target for anti-cancer therapy in HCC. However, before further exploration of this therapeutic strategy, it is critical to understand the clinical relevance of furin over-expression in HCC patients. In this study, we conducted a clinical analysis for the prognosis predictive value of furin expression in HCC patients receiving surgical resection of the tumors. Surprisingly, it was found that over-expression of furin in HCCs predicted a better postoperative disease-free survival (DFS). The growth regulatory effect of furin was further investigated in a xenograft model by use of the furin inhibitor, decRVKR-CMK.

## Materials and Methods

### Patients

Liver tissues from 105 HCC patients, 72 males and 33 females, receiving total removal of liver tumors from 1998 to 2001 in Chang Gung Memorial Hospital were retrieved from Institutional Tissue Bank, Chang Gung Medical Center. Preoperative diagnosis of HCC was made by one of the following methods: echo-guided liver biopsy, fine needle aspiration cytology, high alpha-fetoprotein (AFP) level (>200 ng/mL) plus at least one dynamic imaging studies (dynamic computed tomography or magnetic resonance imaging), or one dynamic imaging studies plus angiography (if AFP<200 ng/mL). Tumors were totally removed with a safety-margin of >1 cm. Post-operative follow-up was performed by ultrasonography, chest X-ray, AFP, and blood biochemistry every 1 to 3 months in the first year and 3 to 6 months thereafter. Abnormal findings were verified by computed tomography or magnetic resonance imaging. Intrahepatic recurrence was verified by use of the aforementioned criteria. Extrahepatic recurrence was verified by biopsy, aspiration cytology, computed tomography or magnetic resonance imaging study dependent on the location of the lesions as well as the condition of the patients. The basic clinicopathological data were retrospectively reviewed: age, cirrhosis, hepatitis B antigen (HBsAg) positive, antibody against hepatitis C (anti-HCV) positive, tumor number, tumor size, ascites, AFP, albumin, bilirubin, prothrombin time, creatinine, aspartate aminotransferase (AST), alanine aminotransferase (ALT), and alcohol usage (Table 1). The study protocol was approved by the Medical Ethics and Human Clinical Trial Committee of the Chang Gung Memorial Hospital (IRB NO 99-3530B). Written informed consent was obtained from all patients, and the study was approved by the Ethics Committee of Chang Gung Memorial Hospital.

**Table 1:** Basic clinical characterization of patients included.

	Gender		
Clinical parameters	Female (n = 33)	Male (n = 72)	P
Age (years)	58.3±13.0	55.4±16.5	0.333
Cirrhosis	19 (57.6%)	34 (47.2%)	0.439
HBsAg positive	17 (51.5%)	56 (77.8%)	0.013
Anti-HCV positive	16 (48.5%)	14 (19.4%)	0.005
Tumor number			
1	24 (72.7%)	43 (59.7%)	0.285^a^
2	4	13	
3	4	13	
4	1	3	
Size (Diameter, cm)	6.9±5.0	6.8±4.7	0.986
Ascites	1 (3.1%)	7 (9.7%)	0.422
Alpha-fetoprotein (ng/mL)	106 (3–327500)^b^	57 (3–125446)^b^	0.522^c^
Albumin (g/dL)	3.6±0.6	3.8±0.7	0.221
Bilirubin (mg/dL)	1.1±1.1	1.5±2.3	0.255
Prothrombin time (sec)	12.3±1.4	12.4±1.4	0.705
Creatinine (mg/dL)	1.1±1.0	1.2±0.8	0.607
AST (U/L)	106.9±120.2	104.1±146.6	0.921
ALT (U/L)	64.9±61.2	89.2±130.3	0.222
Alcoholism	2 (6.1%)	25 (34.7%)	0.004

a Comparison between patients with tumor number  = 1 and those with tumor number >1.

b Median (range).

c Mann-Whitney test.

### Statistical Analysis

Linear regression analysis was performed to estimate the relationship between furin expression (T/N ratio) and clinicopathological variables. The furin expression level was quantified using the Image Gauge software (Fuji Film, Tokyo, Japan) to determine the intensities of the immunoreactive bands as described previously [19]. Disease-free survival was measured from the date of diagnosis to the date of recurrence, metastasis, death or last follow-up. The Kaplan-Meier method was used to compare the survival curves between groups, and the log-rank test was used to estimate the survival probability. To determine the cutoff of furin T/N ratios for survival analysis, experimental Kaplan-Meier analysis was performed using a series of increasing values as the cutoffs. The experimental cutoffs were: The smallest T/N ratio + n/5 × (The largest T/N ratio – The smallest T/N ratio); n = 1 to 4. The multivariate regression was performed to evaluate the joint effect of other factors. Statistical analysis was conducted by use of SPSS version 15.0 (Chicago, IL).

### Reagents and Antibodies

DecRVKR-CMK, a synthetic lipophilic furin inhibitor can penetrate the plasma membrane to reach the interior of cell, where it interacts with furin and blocks its catalytic site by irreversibly binding [20], [21] was purchased from Calbiochem (Darmstadt, Germany). Anti-furin antibody from Affinity BioReagent (Golden, CO) was used for immunoblot and immunostain assay. Anti-Ki-67 antibody from Millipore Corp. (Bedford, MA) was used for immunostain assay. The rabbit polyclonal antibodies to NFκB/p65, CDK2, CDK4, and cyclin D1 from Millipore Corp. and TGFβ1, and Bcl-xL from Cell Signaling Technology, Inc. (Beverly, MA) were used for immunoblot analysis. Mouse monoclonal antibody to insulin receptor (IR), IKKα from Millipore Corp., and GAPDH Chemicon (Bedford, MA) were used for immunoblot.

### Cell Culture and Transfection

Human hepatoma cell line (Huh7) was obtained from American Type Culture Collection (Manassas, VA). A cDNA fragment encoding furin was isolated and inserted into pcDNA3 vector (Invitrogen, Carlsbad, CA) to generated pcDNA3-Furin [18]. Huh7 cells were stably transfected with pcDNA3-Furin using TurboFect (Fermentas, *Life* Technologies, Karlsruhe, Germany) to obtain Huh7-Furin cells. Stable cell colonies were selected by G418 (600 µg/mL) (Amresco USA). Also, Huh7 cells were stably transfected with the empty vector (pcDNA3) to generate Huh7-Neo cells as a mock control. Stable cell lines were maintained in Dulbecco’s modified Eagle’s medium (DMEM) containing 10% fetal calf serum and 600 µg/mL of G418.

### Immunoblot Analysis

Cells and tissues were lysed in lysis buffer (125 mM Tris-phosphate pH 7.8, 10 mM DTT, 10 mM CDTA pH 7.8, 50% glycerol, and 5% Triton-X100). Protein concentration was measured using a Bradford assay kit (Pierce Biotechnology, Rockford, IL). Equal amounts of protein were loaded on a 10% SDS-polyacrylamide gel for electrophoresis before being transferred to a PVDF membrane (PerkinElmer, Boston, MA). The membrane was blotted with rabbit polyclonal antibodies to furin, TGFβ1, NFκB/p65, cyclin D1, Bcl-xL, CDK2, CDK4, and or mouse monoclonal antibody to IR, IKKα, and GAPDH. The blots were incubated with horseradish peroxidase conjugated secondary antibody and developed using an ECL detection kit (Millipore).

### Gelatin Zymography

Cells were treated with 50 µM decRVKR-CMK dissolving in 2.5% DMSO or with 2.5% DMSO only (mock) for two days. To assess the MMP-2 activity, samples with non-denaturing were loaded onto a 10% polyacrylamide gel containing 0.1% gelatin. After electrophoresis, the gels were washed in washing buffer (2.5% Triton X-100), and then incubated overnight at 37°C in the reaction buffer (40 mM Tris-HCl pH 8.0, 10 mM CaCl_2_, and 0.01% NaN_3_). The gels were developed in staining solution (0.1% Coomassie Brilliant Blue R-250, 0.1% amido black, 50% methanol, and 10% acetic acid).

### Animal Model

Five-week-old, male BALB/cAnN.Cg-*Foxnl^nu^*/CrlNarl nude mice were used for subcutaneous injection of Huh7-Neo or Huh7-Furin cells (1×10^6^). The xenografts were allowed to grow until they reached to the size of 40 mm^3^. Intraperitoneal (IP) injection of 300 µL 2.5% DMSO or decRVKR-CMK dissolved in 2.5% DMSO (630 µM) was subsequently performed twice a week for four times. The tumor volume (mm^3^) was measured and calculated using the formula (W^2^ × L)/2 (W, the smallest diameter; L, the longest diameter). All procedures were performed under sterile conditions in a laminar flow hood. The mice experiments were performed in accordance with U.S. National Institutes of Health guidelines, and the Chang Gung Institutional Animal Care and Use Committee Guide for Care and Use of Laboratory Animals. This study was conducted under the approval of Chang Gung Institutionally Animal Care and Use Committee (IACUC Approval NO CGU10-089).

### Immunohistochemistry (IHC)

Paraffin-fixed tumor sections (5- µm thick) were deparaffinized and stained with hematoxylin and eosin. To detect Ki-67 or furin, serial tumor sections were made and IHC was performed as previously described [22]. To verify the specificity of furin signal in IHC staining, recombinant furin protein synthesized using TNT® Quick Coupled Transcription/Translation Systems (Promega) was utilized as a blocking protein for parallel IHC staining control.

### Terminal Uridine Nick-end Labeling (TUNEL) Assay

DNA fragmentation was detected using the DeadEnd Fluorometric TUNEL System assay (Promega, Madison, WI) according to the manufacturer’s instructions. The TdT-mediated dUTP-biotin nick end labeling reaction was performed by use of ﬂuorescein isothiocyanate-dUTP at 37°C for 60 min. Fluorescence of apoptotic cells (green) and of nuclei (blue; stained with DAPI) was detected by fluorescence microscopy (Olympus IX71).

## Results

### Clinicopathological Analysis of Furin Expression in HCC Patients

**Figure 1:**
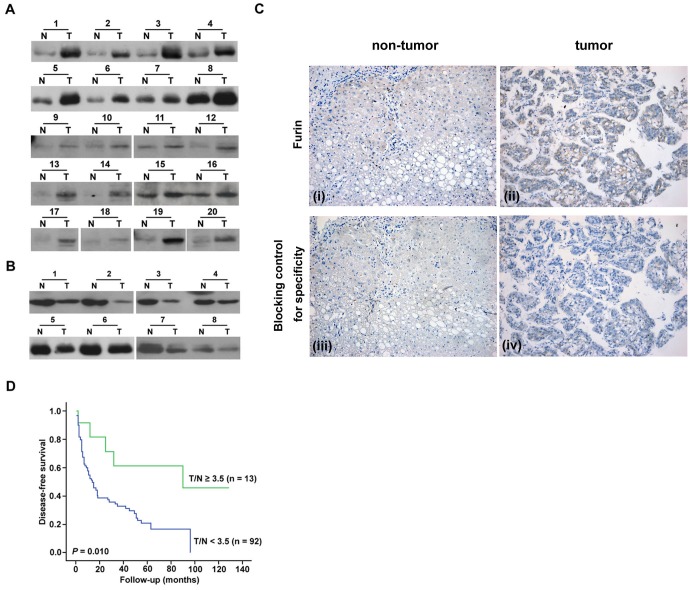
A dual and contradictory role of furin in HCC. (A) Furin overexpression in 20 pairs of representative cancerous (T) and adjacent noncancerous (N) tissues demonstrated by immunoblot analysis. (B) Furin underexpressed in the 8 representative paired HCC tissues. (C) Expression of furin assessed by IHC staining in noncancerous and cancerous liver tissue (i and ii). Specificity of the IHC staining was verified by blocking of the primary antibody with recombinant furin protein (iii and iv). (D) Disease-free survivals in HCC patients expressing different levels of furin (Kaplan-Meier survival analysis).

To understand the clinical relevance of furin expression in HCC, paired cancerous and adjacent noncancerous HCC tissues were obtained from 72 male and 33 female HCC patients. Furin protein expression was higher in the cancerous part compared to that in the non-cancerous part in 81/105 of the tumor tissues (Fig. 1A), whereas it was lower in the cancerous part in the remaining patients (Fig. 1B). Immunohistochemistry showed expression of furin mainly in the hepatocytes in the HCC tissue (Fig. 1C), but rarely in other cellular components.

By testing a series of cutoff values (see Materials and Methods), Kaplan-Meier survival curves and log-rank test indicated that patients with T/N ratios of furin expression ≥ 3.5 (n = 13) in hepatoma tissues had significantly longer DFS compared to those with T/N ratios <3.5 (n = 92) (*P* = 0.010) (Fig. 1D). On the other hand, no significant association between the furin expression and overall survival was found (data not shown). The Cox proportional hazards model was executed to further verify the associations between furin expression and clinicopathological factors for DFS in patients with HCC. Univariate analysis revealed that microvascular invasion, tumor number >1, macrovascular invasion, ascites, AFP>25 ng/mL, albumin ≤ 4.0 g/dL, and furin expression T/N ratio <3.5 were significantly associated with a shorter DFS. However, when these confounding factors were included, multivariate analysis showed that tumor number >1, macrovascular invasion, albumin ≤ 4.0 g/dL, and furin expression T/N ratio <3.5 were independent factors correlated with a shorter DFS (Table S1).

### Generation of Huh7 Cells Stably Expressing Furin

**Figure 2:**
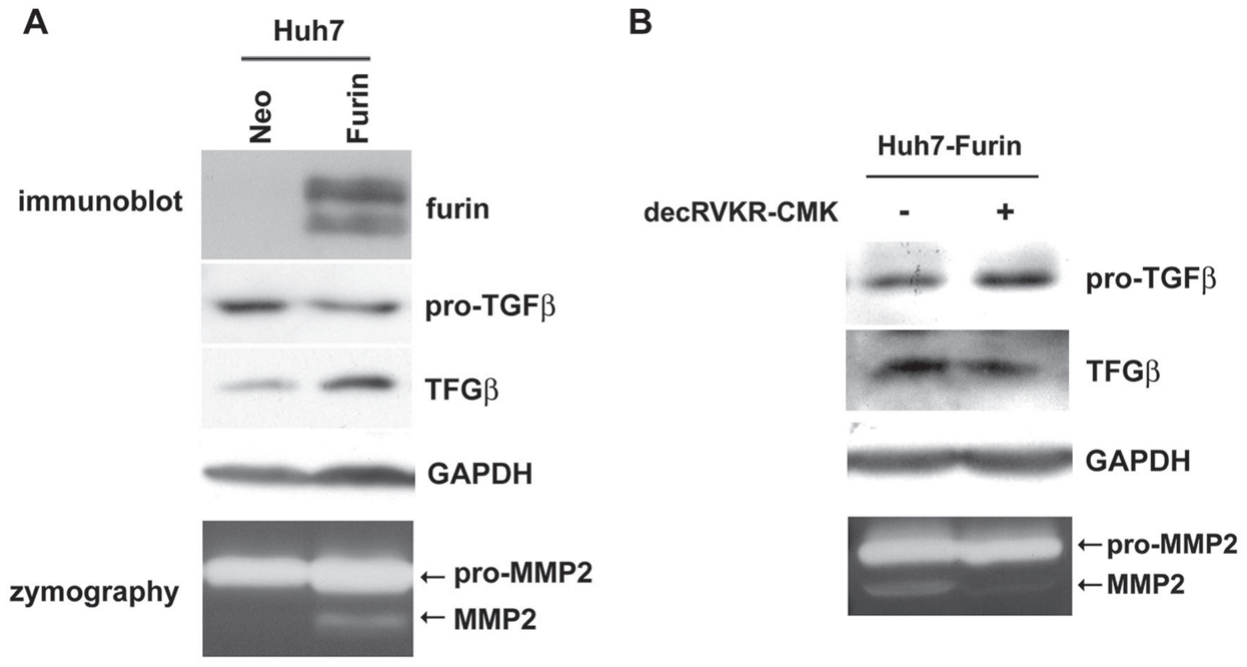
Inhibition of furin activity by decRVKR-CMK in Huh7-Fruin cells. (A) Expression levels of furin, pro-TGFβ1 and TGFβ1 in Huh7-Neo and Huh7-Fruin cells were showed by immunoblot. Gelatin zymography was performed to detect the MMP2 activities. (B) Immunoblot was performed to determine the pro-TGFβ1 and TGFβ1 expression levels and gelatin zymography was performed to determine the MMP2 activities in the absence (-) and presence (+) of decRVKR-CMK.

Previous study indicated that furin was overexpressed in liver cancer and overexpression of furin enhanced invasiveness of HepG2 cells in tail vein xenograft models [18]. Presumably, furin overexpression should be associated with a shorter survival for HCC patients. However, the Kaplan-Meier analysis and proportional hazards model demonstrated that a higher expression level of furin (T/N ratio ≥ 3.5) associated with longer DFS in HCC patients (Fig. 1D and Table S1). To understand the reason why furin over-expression resulted in longer DFS of patients with HCC, the growth regulatory effects of furin when over-expressed in hepatoma cells were examined in a xenograft model. Because subcutaneous tumor formation only occurred in <50% of mice injected with HepG2 cells [23], Huh7 cells were utilized to generate xenografts stably over-expressing furin in this study. This cell line had very low endogenous furin expression [18] and was much easier to form tumors than HepG2 cells in mice. The expression level of furin significantly increased in Huh7-Furin cells compared with Huh7-Neo control cells (Fig. 2A). Two of the furin substrates, TGFβ1 [2], [24] and MMP2 [25], were used for functional analysis. The expression level of pro-TGFβ1 decreased, while that of TGFβ1 increased in Huh7-Furin cells, compared with those in Huh7-Neo cells (Fig. 2A). Zymography assay indicated that active MMP2 was only observed in Huh7-Furin cells but not in the Huh7-Neo cells. A synthetic furin inhibitor for the inhibition of PC activity, decRVKR-CMK, was used in this study. To confirm the inhibition ability of decRVKR-CMK in Huh7-Furin cells, it was found that the amount of pro-TGFβ1 was increased, while that of TGFβ1 was reduced, after Huh7-Furin cells were treated with 50 µM/L decRVKR-CMK. Also, conversion of pro-MMP2 to MMP2 in Huh7-Furin cells was inhibited in the presence of decRVKR-CMK (Fig. 2B).

### Inhibition of Furin Activity by decRVKR-CMK Promoted Tumor Growth in Huh7-Furin Xenograft

**Figure 3:**
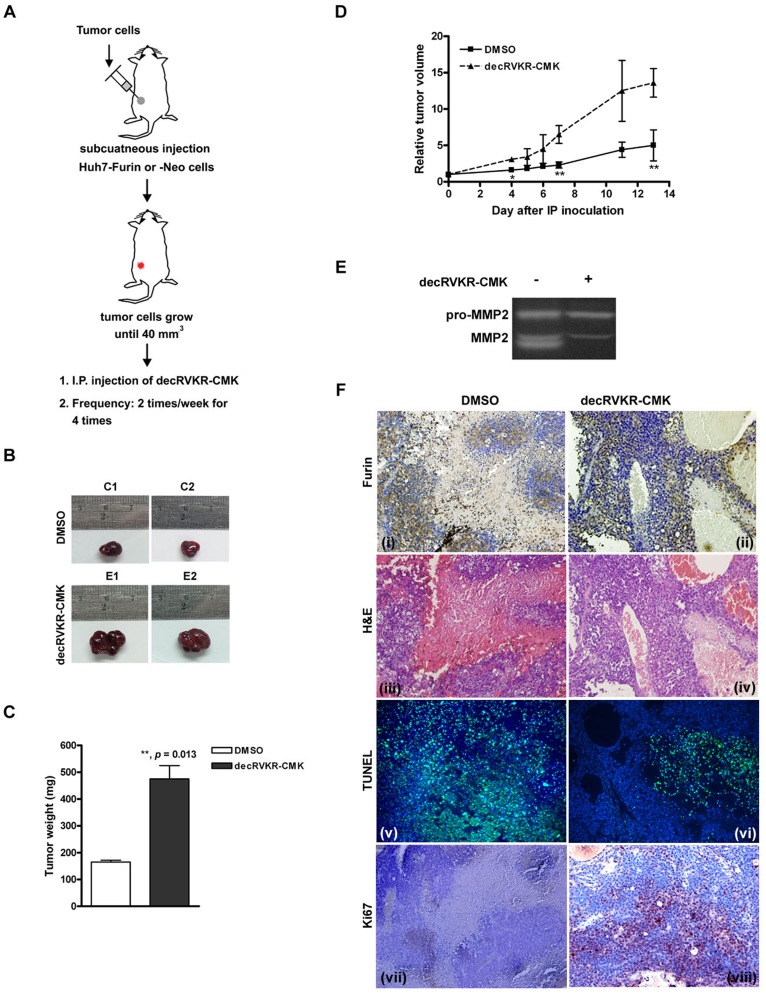
Treatment with decRVKR-CMK promoted cell viability in Huh7-Furin xenograft tumor. (A) Animal experimental protocol was shown. (B to D) Comparisons of the tumor volumes between Huh7-Furin xenografts with or without decRVKR-CMK. (E) Gelatin zymography was performed to detect the MMP2 activities. (F) Immunohistochemistry analysis for xenograft tumor section. Immunostain for furin (i and ii) and Ki-67 (vii and viii); H&E stain (iii and iv); TUNEL assay (v and vi) (200X). The areas marked by yellow dashed lines in (iii) and (iv) were necrosis areas.

**Figure 4:**
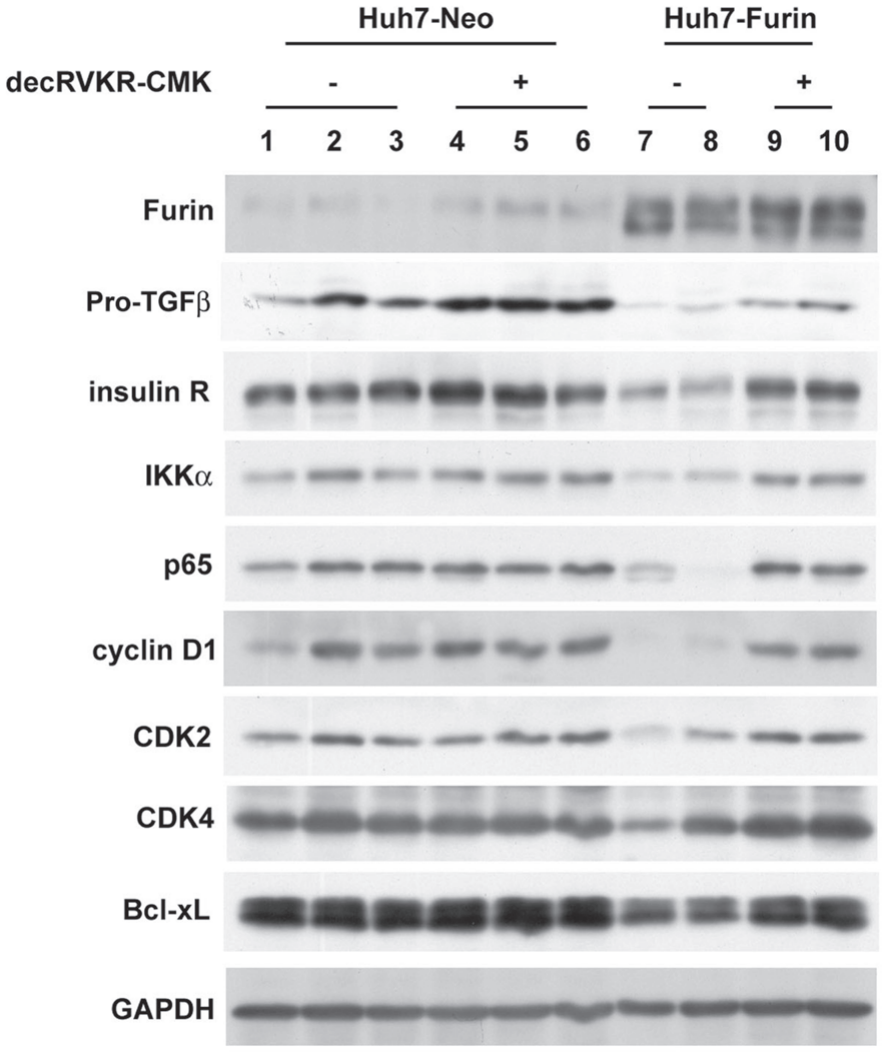
Differential expression of cell proliferation and cell death regulatory molecules. The expression level of furin, pro-TGFβ1, and several cell proliferation and cell death regulatory molecules between Huh7-Neo and Huh7-Furin xenograft tumors after administration with or without decRVKR-CMK was analyzed by immunoblot.

To investigate the growth regulatory effect of over-expressed furin in hepatoma cells, the mice carrying Huh7-Furin xenografts were intraperitoneally (IP) injected with or without decRVKR-CMK. Prior to the injection of inhibitor, the subcutaneous Huh7-Furin xenograft tumors were generated in nude mice. When the tumor size reached to the volume approximately 40 mm^3^, DMSO (as a mock control) or decRVKR-CMK (dissolved in DMSO) was injected through IP route twice a week for four times (Fig. 3A). Four Huh7-Furin xenograft tumors were generated and divided into DMSO (n = 2) and decRVKR-CMK (n = 2) groups. As shown in Fig. 3B and 3C, the final tumor volume and weight of the Huh7-Furin xenografts were significantly increased in decRVKR-CMK group (475.0±49.50 mg) compared with those in DMSO group (165.0±7.07 mg) (*P* = 0.013). In addition, the growth rate of Huh7-Furin xenograft was significantly increased in decRVKR-CMK treated group (Fig. 3D). The level of mature MMP2 was significantly reduced in decRVKR-CMK treated xenograft tumors compared to that in DMSO treated group (Fig. 3E) confirming the inhibitory effect of decRVKR-CMK in the mouse model. Similarly, the pro-TGFβ1 was elevated in decRVKR-CMK treated group (Fig. 4). Additionally, immunohistochemistry and immunoblot analysis in Huh7-Furin xenograft tumors detected equally strong expression of furin in both of the DMSO and decRVKR-CMK treated groups (Figs. 3F, i and ii; Fig. 4), suggesting that the difference of growth rate was caused by functional inhibition but not a different expression level.

### Absence of Growth Regulatory Effect on Huh7-Neo Xenografts with decRVKR-CMK

**Figure 5:**
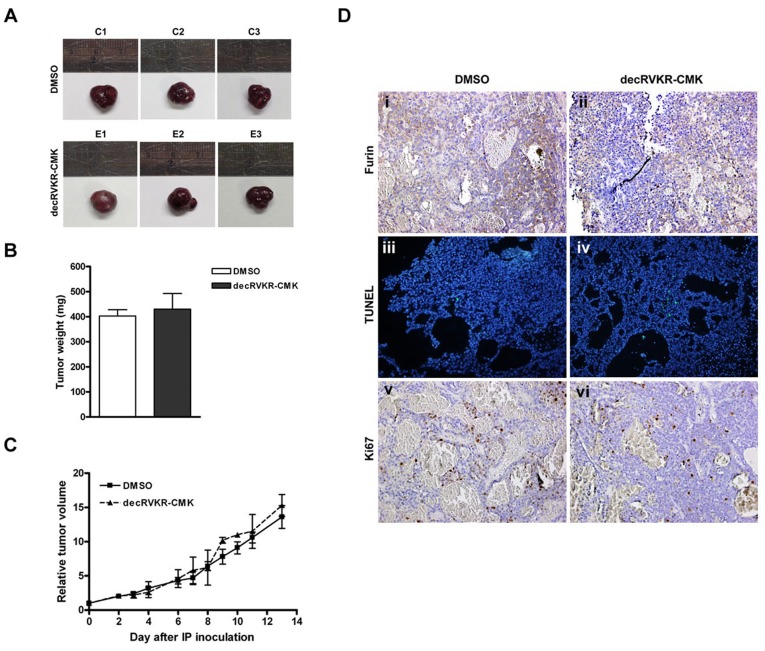
No effects of cell viability by treatment with decRVKR-CMK in Huh7-Neo xenograft tumor. (A to C) Comparisons of the tumor volumes between Huh7-Neo xenografts with or without decRVKR-CMK. (D) Immunohistochemistry analysis for xenograft tumor section. Immunostain for furin (i and ii) and Ki-67 (v and vi); TUNEL assay (iii and iv) (200X).

The procedure of generating subcutaneous Huh7-Neo xenografts and IP injection of decRVKR-CMK was the same as described in the previous section. Six xenograft tumors were generated and divided into DMSO (n = 3) and decRVKR-CMK (n = 3) groups. No significant difference was found for the final tumor volume, tumor weight, and tumor growth rate of Huh7-Neo xenografts between the decRVKR-CMK and DMSO groups (Figs. 5A to 5C). The immunohistochemistry and immunoblot analysis detected equally lower expression of furin in Huh7-Neo xenografts in both of the DMSO and decRVKR-CMK treated groups (Figs. 5D, i and ii; Fig. 4), compared with those in Huh7-Furin xenografts (Figs. 3F, i and ii; Fig. 4). The increase of pro-TGFβ1 expression was observed in decRVKR-CMK treated Huh7-Neo xenografts compared to those in DMSO treated group despite that Huh7 cells expressed very low endogenous furin. However, the overall expression level of pro-TGFβ1 was higher in Huh7-Neo than in Huh7-Furin xenografts (Fig. 4). Moreover, in DMSO treated groups (Figs. 3 and 5), it was found that once the Huh7-Neo xenograft tumor was formed, the tumor weight and tumor growth rate was higher when compared with those in Huh7-Furin xenografts (Figs. 3C versus 5B; Figs. 3D versus 5C), further supporting that furin played a role in repression of tumor growth.

### Alterations of Cell Proliferation and Apoptosis in Huh7-Neo and Huh7-Furin Xenografts treated by decRVKR-CMK

To further analyze the growth-promoting effect of decRVKR-CMK in Huh7-Furin xenografts, histology was examined under H&E stain. A significantly larger necrosis area was observed in DMSO group (mean necrosis area per high power field calculated using a computer program, ImageJ [National Institutes of Health, Bethesda, Maryland, USA]; DMSO versus decRVKR-CMK group, 54.7±4.04% versus 28.8±4.67%, *P*<0.001; Figs. 3F, iii and iv). In addition, TUNEL assay revealed more apoptotic cells in DMSO group (mean percentages of apoptotic cells per high power field, DMSO versus decRVKR-CMK, 73.5±14.11% versus 12.1±3.28%, *P*<0.001; Figs. 3F, v and vi). Finally, Ki-67, a proliferation marker, was detected in more hepatoctyes in decRVKR-CMK treated group (mean percentages of Ki-67-positive cells per high power field, DMSO versus decRVKR-CMK, 21.9±9.82% versus 75.3±5.15%, *P*<0.001; Figs. 3F, vii and viii).

However, in Huh7-Neo xenografts, no difference was observed for the percentages of apoptotic cells and the Ki-67 expression cells between DMSO and decRVKR-CMK treated groups (Figs. 5D, iii versus iv, and v versus vi).

### Reduced Expression of Growth and Anti-apoptotic Regulatory Factors in Huh7-Furin Xenograft

The xenograft experiments revealed poor cell viability in Huh7-Furin xenograft tumor, whereas decRVKR-CMK treatment resulted in improved cell viability and enhanced cell proliferation. Furthermore, better cell viability of Huh7-Neo xenograft tumor (both DMSO and decRVKR-CMK group) was observed compared with that in DMSO treated Huh7-Furin group. To investigate the possible molecular mechanism, several factors involved in IGF, PI3K/AKT, EGFR/RAS and Wnt mediated signaling were examined, which were presumably up-regulated during hepatocarcinogenesis [26]–[29]. As shown in Fig. 4, immunoblot analysis revealed an under-expression level of insulin receptor, IKKα, and NFκB/p65 in DMSO treated Huh7-Furin xenograft compared with those in Huh7-Neo xenograft. Upon decRVKR-CMK administration, the expression levels of these factors restored significantly in Huh7-Furin xenografts. Previous reports indicated that increased expression in CDK2, CDK4, and cyclin D1 were found in HCC [28], [30], besides their associations with cell cycle progression [31]. The expression levels of these factors were also found to be reduced in DMSO treated Huh7-Furin xenografts compared with those in Huh7-Neo groups and restoration of these factors was also observed in decRVKR-CMK treated Huh7-Furin group. Similar results were found in Bcl-xL, an anti-apoptotic protein which was over-expressed in HCC [32]. Furthermore, TGFβ1 could down-regulate the expression of CDK4 and Bcl-xL [33], [34], even when cell cycle was arrested at G1 phase [35]. As TGFβ1 was hard to detect in xenograft tumor, the expression level of pro-TGFβ1 was presented. The increased levels of pro-TGFβ1 implying decreased level of TGFβ1 in decRVKR-CMK treated Huh7-Furin xenografts.

## Discussion

Many studies indicated that furin, a pro-protein convertase, is over-expressed in human cancer cell lines and primary malignancies [8]–[13]. Some researches further suggested that furin could be a candidate molecular target for anti-cancer therapeutics [14], [17]. Recently, it was reported that furin overexpression also occurred in human HCCs [18]. In addition, overexpression of furin in hepatoma cells resulted in increased invasiveness in tail vein xenograft model [18]. To investigate whether furin could be a candidate target for anti-liver cancer therapy, the association between furin expression and clinicopathologic parameters in HCC patients was analyzed. The Kaplan-Meier survival analysis and Cox regression analysis indicated that a higher expression level of furin (T/N ratios ≧ 3.5) in hepatoma tissues associated with longer DFS. The clinicopathological analysis implied that furin inhibition in hepatoma tissues in which furin was over-expressed might result in worse prognosis in HCC patients and furin might not be a proper target for anti-liver cancer therapy.

Despite the unfavorable clinical data, increased tumorigenicity of furin has been suggested in an *in vivo* study. In *PLAG1* overexpressed mice which promoted adenomas occurrence in salivary glands, simultaneous furin deficiency resulted in delayed tumorigenesis [36]. To clarify these puzzles, subcutaneous Huh7-Neo and Huh7-Furin xenograft tumors were generated and furin inhibitor (decRVKR-CMK) was administrated after the tumors grew to a comparable size. In this assay, no significant difference of tumor growth was found between DMSO and decRVKR-CMK treated groups in Huh7-Neo xenografts. However, the tumor growth rate was slower in DMSO treated than that in decRVKR-CMK treated Huh7-Furin xenografts. Interestingly, once the Huh7-Neo xenograft tumors (DMSO and decRVKR-CMK groups) were formed, the growth rate is faster than DMSO treated Huh7-Furin xenografts. Pro-TGFβ1 is a substrate of furin, of which the active form (TGFβ1) suppresses the growth of Hep3B and Huh7 hepatoma cells [37]. The decrease of pro-TGFβ1 expression in Huh7-Furin xenografts, implying the increase of TGFβ1, might explain the growth inhibition effects of over-expressing furin. In addition, involvement of furin in repression of tumor growth was also supported by decreased expression of cell proliferation related molecules (IR, cyclin D1, CDK2, and CDK4…etc.). Down-regulation of CDK4 by TGFβ1 has also been reported [34]. Thus, inhibition of CDK4 expression in Huh7-Furin xenografts might be mediated through TGFβ1. Furthermore, the repression of tumor growth was restored when furin inhibitor was utilized in Huh7-Furin xenograft, whereas no growth regulatory effect was observed when furin inhibitor was administrated to Huh7-Neo xenografts. The protein expression levels of growth related molecules were increased and stronger Ki-67 expression was detected in decRVKR-CMK treated Huh7-Furin xenografts. Furthermore, the increased levels of these molecules were similar to those in Huh7-Neo xenografts, indicating a restoration of the growth inhibition effect by furin. These data were consistent with the clinical observation that furin over-expression with a T/N ratios ≧ 3.5 associates with a longer DFS in HCC patients.

In addition to the growth effect of furin, the alteration of cell apoptosis was also examined. H&E stain revealed a larger necrosis area, and TUNEL assay detected more apoptotic cells in the decRVKR-CMK untreated Huh7-Furin tumors, which were reversed upon decRVKR-CMK treatment. The expression levels of cell death related factors such as IKKα, NFκB/p65 and Bcl-xL were reduced in Huh7-Furin tumor and were restored after decRVKR-CMK administration. Besides growth inhibition, TGFβ1 also induced apoptosis in hepatoma cell [37]. Furthermore, down-regulation of Bcl-xL expression by TGFβ1 was reported [33].

The relationship between furin and patient survival in this study was based on comparison of the T/N ratios of furin expression levels. One might argue that in patients with high T/N ratios, a relatively higher level of furin in the non-cancerous (N) parts was more important than those in the cancerous (T) parts. However, all our previous and present results regarding the growth regulatory roles of furin in HCC were derived from experiments performed in hepatoma (transformed) cells but not immortalized, non-cancerous cells. Therefore, it is unclear whether a high furin expression level in the noncancerous cells plays a role in inhibition of de novo cancer growth. In this study, we assumed that most of the postoperative HCC recurrences were originated either from micro-spread of the original cancer or from de novo carcinogenesis utilizing a similar oncogenic mechanism. As such, the furin T/N ratios derived from the surgically removed HCC were used to correlate with recurrence. Furthermore, when we examined some patients with high T/N ratios of furin but a generally low expression level of furin in the non-cancerous part, a long disease-free survival remained.

In our previous study, furin was found to promote HepG2 cells invasion in tail vein xenograft model [18]. The results motivate us to investigate whether inhibition of furin activity could be a novel therapeutic approach for liver cancer therapy. However, when analyzing relationship between furin expression and DFS in HCC patients, we discovered that over-expression of furin associated with longer DFS. Thus the growth regulatory effects of furin in hepatoma xenografts were focused in this study. Using the subcutaneous xenograft model, the growth inhibitory function of furin was discovered. Decreased level of pro-TGFβ1 was observed in furin over-expressed xenografts. It has been reported that TGFβ1 functions in suppressing proliferation and promoting invasion of cancer cells, indicating that TGFβ1 can serve as a tumor suppressor and a pro-metastatic factor [35]. The dual role of TGFβ1 may explain our observations that furin enhances hepatoma cell invasiveness in the tail vein xenograft model, while suppresses tumor growth in the subcutaneous xenograft model. Actually, another PC family member, PC5/6, possessing similar function but not conducive to tumor growth was reported using PC5/6 intestine-specific knockout mice (PC5/6 iKO) [38]. It was found that when Apc^Min/+^ mice which spontaneously develop polyps in small intestine [39] lacking PC5/6 tend to form higher tumor number than non-PC5/6 deficient Apc^Min/+^ mice [38].

In conclusion, over-expression of furin in xenograft tumor in fact exerted a growth inhibitory effect and this effect could be reversed by decRVKR-CMK treatment.

## Supporting Information

Table S1 **Univariate and multivariate analysis of clinicopathological parameters for disease-free survival in HCC patients.**
(DOC)
