# Supplementary material for: Furin Overexpression Suppresses Tumor Growth and Predicts a Better Postoperative Disease-Free Survival in Hepatocellular Carcinoma
Source: PLoS One. 2012 Jul 10;7(7):e40738. doi: 10.1371/journal.pone.0040738 (PMC3393699; doi:10.1371/journal.pone.0040738)
Supplement: Table S1 — Univariate and multivariate analysis of clinicopathological parameters for disease-free survival in HCC patients. (DOC) [file pone.0040738.s001.doc]

**Table S1.** Univariate and multivariate analysis of clinicopathological parameters for disease-free survival in HCC patients.

| Parameter |  | No. of patients | Mean disease-free survival, months (95% CI) | HR (95% CI) | Adjusted HR (95% CI) |
| --- | --- | --- | --- | --- | --- |
| Age (years) | ≦ 60 | 57 | 38.4 (25.7 – 51.1) |  |  |
|  | ＞ 60 | 48 | 36.4 (24.9 – 48.0) | 0.948 (0.584 – 1.538) |  |
| Gender | Female | 33 | 41.2 (26.1 – 62.3) |  |  |
|  | Male | 72 | 36.0 (25.2 – 46.8) | 1.174 (0.691 – 1.993) |  |
| Cirrhosis | No | 52 | 44.6 (29.5 – 59.7) |  |  |
|  | Yes | 53 | 31.6 (21.6 – 41.5) | 1.237 (0.769 – 1.990) |  |
| Alcoholism | No | 78 | 39.6 (28.3 – 50.9) |  |  |
|  | Yes | 27 | 38.2 (20.7 – 55.7) | 1.037 (0.612 – 1.759) |  |
| **Tumor characteristics** | |  |  |  |  |
| Microvascular invasion | No | 74 | 49.0 (36.6 – 61.5) |  |  |
|  | Yes | 31 | 14.2 (6.9 – 21.5) | 2.832 (1.713 – 4.681)^a^ | 1.299 (0.725 – 2.329) |
| Edmondson’s grading | I-II | 28 | 39.1 (23.2 – 55.0) |  |  |
|  | III-IV | 77 | 41.1 (28.9 – 53.3) | 1.121 (0.659 – 1.907) |  |
| Encapsulation | No | 28 | 23.3 (10.7 – 35.8) |  |  |
|  | Yes | 77 | 45.0 (32.9 – 57.1) | 0.628 (0.370 – 1.068) |  |
| Tumor number | 1 | 67 | 53.6 (40.3 – 66.9) |  |  |
|  | ＞ 1 | 38 | 12.7 (7.7 – 17.7) | 3.385 (2.048 – 5.596)^b^ | 2.488 (1.397 – 4.430)^h^ |
| Largest tumor size (diameter, cm) | ≦ 3 | 23 | 37.4 (25.6 – 49.2) |  |  |
|  | ＞ 3 | 82 | 35.4 (25.0 – 45.7) | 1.611 (0.861 – 3.012) |  |
| Macrovascular invasion | No | 96 | 42.3 (31.5 – 53.1) |  |  |
|  | Yes | 9 | 18.8 (1.5 – 40.6) | 2.515 (1.232 – 5.136)^c^ | 2.882 (1.305 – 6.367)^i^ |
| Ascites | No | 96 | 40.1 (29.9 – 50.2) |  |  |
|  | Yes | 9 | 6.3 (2.5 – 10.1) | 3.529 (1.567 – 7.947)^d^ | 1.848 (0.782 – 4.367) |
| **Serology** |  |  |  |  |  |
| AFP (ng/mL) | ≦ 25 | 27 | 60.7 (39.1 – 82.4) |  |  |
|  | ＞ 25 | 78 | 34.0 (23.6 – 44.4) | 2.134 (1.129 – 4.034)^e^ | 1.278 (0.643 – 2.540) |
| Albumin (g/dL) | ≦ 4.0 | 67 | 31.6 (19.4 – 43.7) |  |  |
|  | ＞ 4.0 | 38 | 49.6 (34.9 – 64.3) | 0.568 (0.345 – 0.935)^f^ | 0.584 (0.345 – 0.988)^j^ |
| Bilirubin (mg/dL) | ≦ 1.2 | 73 | 44.8 (31.6 – 57.9) |  |  |
|  | ＞ 1.2 | 32 | 32.2 (19.0 – 45.4) | 1.303 (0.794 – 2.137) |  |
| Prothrombin time (sec) | ≦ 12 | 46 | 42.5 (26.3 – 58.6) |  |  |
|  | ＞ 12 | 59 | 38.1 (26.4 – 49.7) | 1.034 (0.639 – 1.673) |  |
| Creatinine (mg/dL) | ≦ 1.0 | 54 | 32.6 (21.5 – 43.6) |  |  |
|  | ＞ 1.0 | 51 | 48.3 (32.3 – 64.2) | 0.748 (0.465 – 1.205) |  |

**Table S1.** Continued

| Parameter |  | No. of patients | Mean disease-free survival, months (95% CI) | HR (95% CI) | Adjusted HR (95% CI) |
| --- | --- | --- | --- | --- | --- |
| AST (U/L) | ≦ 36 | 34 | 44.8 (27.3 – 62.3) |  |  |
|  | ＞ 36 | 71 | 36.0 (24.8 – 47.3) | 1.414 (0.840 – 2.383) |  |
| ALT (U/L) | ≦ 25 | 22 | 30.5 (20.2 – 40.7) |  |  |
|  | ＞ 25 | 83 | 38.7 (27.8 – 49.6) | 1.198 (0.651 – 2.204) |  |
| Anti-HCV | Negative | 75 | 42.1 (30.6 – 53.5) |  |  |
|  | Positive | 30 | 24.5 (13.9 – 35.2) | 1.362 (0.789 – 2.350) |  |
| HBsAg | Negative | 32 | 44.0 (24.2 – 63.8) |  |  |
|  | Positive | 73 | 37.1 (26.2 – 48.0) | 1.180 (0.682 – 2.043) |  |
| Furin T/N ratio | Low | 92 | 31.2 (23.4 – 38.9) |  |  |
|  | High | 13 | 79.2 (47.7 – 110.6) | 0.306 (0.118 – 0.796)^g^ | 0.333 (0.120 – 0.925)^k^ |

^a^P < 0.001; ^b^P < 0.001; ^c^P = 0.011; ^d^P = 0.002; ^e^P = 0.020; ^f^P = 0.026; ^g^P = 0.015

^h^P = 0.002; ^i^P = 0.009; ^j^P = 0.045; ^k^P = 0.035

Otherwise, P > 0.05; HR, hazard ratio; CI, confidence interval
